# Immediate versus Delayed Wound Closure in Hidradenitis Suppurativa Surgery: A Comparative Outcomes Study

**DOI:** 10.1055/s-0045-1802326

**Published:** 2025-01-31

**Authors:** Ehud Fliss, Gon Shoham, Tariq Zoabi, Ariela Hafner, Benjamin Meilik, Sharon Manheim, Daniel J. Kedar, Yoav Barnea, Eyal Gur, Eran Otremski

**Affiliations:** 1 Department of Plastic and Reconstructive Surgery, Tel Aviv Sourasky Medical Center, Faculty of Medicine, Tel Aviv University, Tel Aviv, Israel; 2 Department of Dermatology, Tel Aviv Sourasky Medical Center, Faculty of Medicine, Tel Aviv University, Tel Aviv, Israel

**Keywords:** hidradenitis suppurativa, immediate repair, secondary intention

## Abstract

**Background** Hidradenitis suppurativa (HS) is a chronic inflammatory skin disease affecting approximately 1% of young adults. Severe and refractory disease commonly requires surgical excision of the affected skin. To date, there is no consensus regarding the most appropriate reconstructive algorithm.

**Materials and Methods** We conducted a retrospective cohort study including all HS patients who underwent surgical excision in the framework of our multidisciplinary clinic. Operative data and postoperative outcome measures were compared between patients who underwent immediate versus delayed reconstruction. Additionally, reconstructive methods were compared and risk factors for adverse postoperative outcome were identified.

**Results** A total of 103 patients underwent 158 surgeries for HS excision. The overall complication rate was significantly higher in patients who underwent immediate versus delayed wound closure (31 vs. 16%, *p* = 0.039). Any intervention for wound closure (immediate or delayed) was associated with increased risk of postoperative complications in comparison to secondary healing (33 vs. 4%, *p* < 0.001). With delayed closure, the average time to wound closure was 85.4 days with secondary healing only and 57 days with negative pressure wound therapy assisted closure.

**Conclusion** Risk factors for adverse postoperative outcome in HS surgery are multifactorial and involve both timing and method of reconstruction in addition to various patient factors. The findings of this study strengthen the notion that delayed closure of post-HS excision wounds leads to the most uneventful course in regard to postoperative adverse events; however, this may take up to 3 months. Upon deciding on a reconstructive plan, the risk-to-benefit ratio should be assessed individually weighing the pros and cons of immediate closure and delayed secondary intention.

## Introduction

Hidradenitis suppurativa (HS) is a chronic, recurrent, inflammatory skin condition that affects approximately 1% of young adults. It is characterized by flare-ups in which subcutaneous lesions become painful, inflamed, and suppurative. Ultimately, affected regions develop chronic sinuses, nodules, and scarring. The disease is known for dramatically affecting quality of life (QOL) and commonly results in a feeling of low general health. 1 2 3 4 5 Treatment is optimally performed in a multidisciplinary approach and treatment strategy is determined according to multiple parameters including the Hurley stage, the number of affected regions, disease impact on QOL, and patient preferences ( Table 1 ). Localized disease is usually treated with topical treatment and minor interventions, while more widespread and/or severe disease is usually treated with systemic anti-inflammatory antibiotics, biologic immunomodulators, or immunosuppressive drugs. In patients with a Hurley stage 3 or refractory disease, surgical excision offers the best chance for disease control. 1 2 3 6

**Table 1:** Hurley stages of hidradenitis suppurativa

Stage	Characteristics
1	Formation of single or multiple abscesses without sinus tracts and scarring
2	Recurrent abscesses, with sinus tract formation and scarring, occurring as either single lesions or multiple widely separated lesions
3	Diffuse involvement of the affected region, with multiple interconnected tracts and abscesses across the entire area

Tissue defect following HS excision is highly variable in regard to size and location, and various options for reconstruction exist. The literature regarding reconstruction following HS excision is vast and has been growing steadily; however, no consensus exists regarding the most appropriate form of reconstruction. Moreover, many of the publications on the matter are descriptive in nature, and comparative outcome studies are still lacking.

Patients treated in our multidisciplinary clinic have been generally treated by one of two methods: immediate reconstruction (via primary closure or skin grafting) or delayed reconstruction (via delayed surgical closure or secondary intention with or without negative pressure wound therapy [NPWT]). The aim of this study is to describe these cohorts and compare operative and postoperative outcome measures according to both timing and method of reconstruction. Additionally, risk factors for adverse postoperative outcome shall be identified.

## Materials and Methods

### Patients and Study Design

After receiving approval by our local institutional review board, we performed a retrospective cohort study. We retrieved data on all patients who underwent surgery for HS excision during the years 2015 and 2021 and had at least 3 months of follow-up. All cases were managed by the senior author. Patients whose photographs are presented in the article have granted informed consent.

Demographic data included age, gender, comorbidities, smoking within 1 month prior to surgery, preoperative body mass index (BMI), Hurley stage, prior or current biologic treatment (more than or within 2 weeks prior to surgery, respectively) and duration of follow-up. Surgical details included operative site and number of operative sites treated during the index surgery, resection area in square centimeters, reconstructive method, and timing of wound closure. Excisional surgery was aimed at achieving a 1- to 2-cm margin of macroscopically normal-appearing skin around the affected area, which is considered the gold standard. 7

Immediate wound closure was defined as wound closure during the index surgery. This was performed by either primary repair (tension-free layered closure) or meshed split-thickness skin graft (STSG). Flaps were not used in this cohort. Delayed wound closure was defined as any method of wound closure other than immediate and included delayed STSG, delayed primary closure, or secondary intention, with or without NPWT. When only secondary intention was used, this was done using foam and alginate dressings. Postoperative data included hospitalization length, complications (hematoma, surgical site infection [SSI], seroma, wound breakdown, and deep vein thrombosis), local recurrence (recurrent suppurative lesions within previous excision site), and reoperations. Wound breakdown was defined as wound dehiscence in cases of primary closure and as partial graft or wound slough in cases of STSG or secondary intention.

### Statistical Analysis

We used several statistical methods to describe the collected data, *p* -value calculated when comparing subcohorts, with values of *p* < 0.05 considered significant. Fisher's exact test was used to quantify the association demographic and operative features and overall complication rate based on the calculated *p* -values. Statistical analyses were performed using SciPy 8 (The SciPy community, version 1.8). Missing data included data regarding total surface area of resection (cm ^2^ ). This was missing in less than 5% and was filled with mean values. For odds ratio (OR) calculation, we used threshold values in conversion to binary data (BMI = 25, age = 40 years, resection area= resection area average + one standard deviation [SD]).

## Results

### Univariate Analysis

A total of 103 patients underwent surgery for HS excision in 158 surgical sites ( Table 2 ). The majority of patients underwent immediate closure ( *n* = 91, 58%), most commonly via primary closure ( *n* = 82, 90%). Within the delayed closure group, the majority of patients underwent NPWT-assisted closure ( *n* = 37, 55%), most commonly as a bridge to STSG. Mean time to wound closure in the delayed closure cohort was 57 days with NPWT and 85.4 days with secondary intention only. A comparison of the immediate versus delayed closure cohorts found that patients who underwent delayed closure had a statistically significant higher mean BMI ( *p* = 0.018), higher rate of Hurley stage 3 ( *p* = 0.001), higher rate of perioperative biologic treatment ( *p* = 0.017), and higher rate of axillary and perineal surgery ( *p* < 0.001 and 0.002, respectively).

**Table 2:** Patient demographics and operative data, according to study groups

	Total, *N* (%)	Immediate closure, *N* (%)	Delayed closure, *N* (%)	*p* -value
**Patients**	103 (100)	60 (58)	49 (42)	
• Number of surgical sites	158 (100)	91(58)	67(42)	
• Surgeries with multiple sites	43 (42)	23 (38)	20 (41)	0.375
• Gender (male)	89 (56)	46 (51)	43 (64)	0.089
• Age at surgery (y)	36.8 ± 13.4	37.6 ± 14.3	35.6 ± 12.1	0.346
• BMI at surgery (kg/m ^2^ )	26.8 ± 4.9	26 ± 4.8	27.9 ± 4.8	0.018 a
**Surgical sites**	158 (100)	91 (58)	67 (42)	
• Groin • Axilla • Perineal • Buttocks • Breast • Other	61 (39) 52 (33) 15 (9) 12 (8) 8 (5) 10 (6)	38 (42) 18 (20) 8 (9) 12 (13) 8 (9) 7 (8)	23 (34) 34 (51) 7 (10) 0 (0) 0 (0) 3 (4)	0.346 <0.001 a 0.002 a 0.728 0.013 a 0.415
**Hurley stage**				
• 1 • 2 • 3	9 (6) 59 (37) 90 (57)	8 (9) 41 (45) 42 (46)	1 (1) 18 (27) 48 (72)	0.001 a
Follow-up (mo)	11.6 ± 14.1	14.4 ± 15.5	7.9 ± 11.1	0.004 a
MDT duration (mo)	26 ± 19.2	28.5 ± 19	22.8 ± 19.1	0.063
Prior biological treatment	77 (49)	39 (43)	38 (57)	0.086
Current biological treatment	52 (33)	23 (25)	29 (43)	0.017 a
Smoking	92 (58)	52 (57)	40 (60)	0.749
Comorbidities b	69 (44)	37 (41)	32 (48)	0.138
Total size of resection (mean), cm ^2^	48.1 ± 64.7	49 ± 70.3	46.8 ± 56.7	0.828
** Reconstruction method c**				
• Primary closure • STSG • Secondary intention only • NPWT-assisted closure	85 (54) 27 (17) 30 (19) 37 (23)	82 (90) 9 (10) 0 (0) 0 (0)	3 (4) 18 (27) 30 (45) 37 (55)	0.0 a 0.005 a 0.0 a 0.0 a
**Time to wound closure within delayed closure cohort (d), mean ± SD**				
• Total • Secondary intention only ( *n* = 30) • NPWT-assisted closure ( *n* = 37) – NPWT only ( *n* = 16) – Delayed surgical closure ( *n* = 21) ○ NPWT > STSG ( *n* = 18) ○ NPWT > primary closure ( *n* = 3)	N/A	N/A	64.2 ± 52.3 85.4 ± 50 57 ± 58 105 ± 60 27.6 ± 32 22.5 ± 25.9 58.3 ± 45.2	N/A
Length of hospital stay (d)	10.2 ± 16.6	8.1 ± 14.8	13.2 ± 18.4	0.053

Abbreviations: BMI, body mass index; MDT, multidrug therapy; N/A, not applicable; NPWT, negative pressure wound therapy; SD, standard deviation; STSG, split-thickness skin graft.

a Statistically significant.

b One or more of the following: inflammatory bowel disease, diabetes mellitus, asthma, dyslipidemia, hypertension, ischemic heart disease, rheumatoid arthritis, and familial Mediterranean fever.

c Numbers represent total reconstructive methods used; patients underwent a combination of methods (e.g., NPWT as a bridge for STSG).

Overall, 39 of the surgical sites (25%) suffered at least one complication ( Table 3 ). The rate of overall complications was significantly higher in the immediate versus delayed closure cohort (31 vs. 16%, *p* = 0.039), with the rate of wound breakdown being significantly higher in the immediate closure group (24 vs. 3%, *p* < 0.001). The overall rate of reoperation due to recurrence was 20%, with a nonstatistically significant higher rate in the immediate closure cohort (23 vs. 16%, *p* = 0.306).

**Table 3:** Postoperative outcome of the patients, according to study groups

	Total	Immediate closure	Delayed closure	*p* -Value
Any complication	39 (25%)	28 (31%)	11 (16%)	0.039 a
Hematoma	5 (3%)	2 (2%)	3 (4%)	0.422
Surgical site infection	11 (7%)	8 (9%)	3 (4%)	0.295
Seroma	2 (1%)	2 (2%)	0 (0%)	0.225
Wound breakdown	24 (15%)	22 (24%)	2 (3%)	0.0 a
Deep vein thrombosis	0 (0%)	0 (0%)	0 (0%)	N/A
Other	8 (5%)	6 (7%)	2 (3%)	0.31
Acute revision	2 (1%)	2 (2%)	0 (0%)	0.225
Late revision (scar revision/other)	15 (9%)	10 (11%)	5 (7%)	0.458
Reoperation for recurrence	32 (20%)	21 (23%)	11 (16%)	0.306

a Statistically significant.

### Multivariate Analysis

Potential risk factors and their OR for various outcome measures were identified for the general cohort ( Table 4 ) and for the two cohorts of immediate and delayed closures ( Table 5 ). In the general study population, several factors were found to be statistically significant risk factors for any complication. These included age above 40 years at surgery (OR: 2.53, *p* = 0.02), total area of resection above the mean + SD (OR = 4.8, *p* < 0.001), and immediate closure versus delayed closure (OR = 2.26, *p* = 0.04). Perioperative biologic treatment was associated with increased risk of hematoma (OR = 8.75, *p* = 0.04) and SSI (OR = 3.97, *p* = 0.04).

**Table 4:** Risk factors for reoperation and postoperative complications in the general study population

	Reoperation for recurrence (OR/ *p* -Value)	Late revision (OR/ *p* -Value)	Complications (OR/ *p* -Value)	Hematoma (OR/ *p* -Value)	SSI (OR/ *p* -Value)	Dehiscence (OR/ *p* -Value)
**General cohort (** ***N*** ** = 158)**						
Age >40 y at surgery	0.46/0.1	0.93/1.0	2.53/0.02 a	1.26/1.0	2.4/0.19	1.41/0.49
BMI >25 at surgery	0.7/0.43	0.24/0.03 a	0.74/0.46	1.14/1.0	0.61/0.53	1.31/0.66
Male gender	1.0/1.0	2.29/0.18	1.53/0.27	0.51/0.65	1.39/0.76	0.9/0.83
Multiple regions	1.78/0.22	2.29/0.26	1.27/0.57	N/A/0.16	2.54/0.33	0.71/0.49
Total size of resection (cm ^2^ )	0.4/0.37	2.89/0.1	4.8/0.0 a	5.0/0.12	4.68/0.03 a	2.86/0.09
Prior biological treatment	2.84/0.02 a	8.02/0.0 a	1.99/0.1	4.38/0.2	5.23/0.03 a	1.29/0.66
Current biological treatment	0.91/1.0	1.41/0.57	1.39/0.43	8.75/0.04 a	3.97/0.04 a	0.64/0.48
Smoking	1.06/1.0	1.08/1.0	1.39/0.46	0.47/0.65	1.28/0.76	1.91/0.19
Comorbidities b	2.55/0.03 a	3.72/0.05	1.89/0.18	0.0/0.55	2.67/0.23	1.12/0.8
Wound closure: immediate vs. delayed	1.53/0.32	1.53/0.59	2.26/0.04 a	0.48/0.65	2.06/0.36	10.36/0.0 a

Abbreviations: BMI, body mass index; OR, odds ratio; SSI, surgical site infection.

Note: OR and *p* -value were calculated using the SciPy.stats module for Python.

a Statistically significant.

b One or more of the following: inflammatory bowel disease, diabetes mellitus, asthma, dyslipidemia, hypertension, ischemic heart disease, rheumatoid arthritis, and familial Mediterranean fever.

**Table 5:** Risk factors for reoperation and postoperative complications according to study group a

	Reoperation for recurrence (OR/ *p* -Value)	Late revision (OR/ *p* -Value)	Complications (OR/ *p* -Value)	Hematoma (OR/ *p* -Value)	SSI (OR/ *p* -Value)	Dehiscence (OR/ *p* -Value)
**Immediate wound closure cohort (** ***N*** ** = 91)**						
Prior biological treatment	3.6/0.02 b	6.45/0.02 b	2.32/0.11	N/A/0.18	11.16/0.02 b	1.87/0.22
Current biological treatment	0.63/0.57	2.18/0.27	0.98/1.0	N/A/0.06	6.02/0.02 b	0.83/1.0
**Delayed wound closure cohort (** ***N*** ** = 67)**						
Age at surgery	0.0/0.01 b	1.51/0.65	5.25/0.03 b	0.0/0.55	1.1/1.0	2.25/0.53
Total size of resection (cm ^2^ )	0.0/0.58	3.62/0.33	31.43/0.0 b	0.0/1.0	0.0/1.0	15.25/0.14
Current biological treatment	1.72/0.51	0.86/1.0	4.44/0.05 b	2.74/0.57	2.74/0.57	1.32/1.0
Delayed wound closure: intervention vs. secondary intention	0.43/0.48	1.51/0.65	16.5/0.0 b	4.74/0.23	4.74/0.23	N/A/0.09

Abbreviations: BMI, body mass index; OR, odds ratio; SSI, surgical site infection.

Note: OR and *p* -value were calculated using the SciPy.stats module for Python.

a Statistically significant data presented only. For complete data please refer to Supplementary tables 2 and 3 , available in the online version.

b Statistically significant.

In the immediate closure cohort, perioperative biologic treatment was associated with increased risk of SSI (OR = 6.02, *p* = 0.02), and prior biologic treatment was associated with increased risk of reoperation due to recurrence (OR = 3.6, *p* = 0.02), late revision (OR = 6.45, *p* = 0.02), and SSI (OR = 11.16, *p* = 0.02). In the delayed closure group, several factors were associated with increased risk of any postoperative complication and included age above 40 years at surgery (OR = 5.25, *p* = 0.03), total area of resection over the mean + SD (OR = 31.43, *p* < 0.001), perioperative biologic treatment (OR = 4.44, *p* = 0.05), and late intervention (i.e., delayed primary closure or STSG) versus secondary intention only (OR = 16.5, *p* < 0.001). Further analysis found increased rate of complications when any intervention was performed compared with secondary intention only (33 vs. 4%, *p* < 0.001). A comparison of the patients according to the Hurley stage found that Hurley stage 3 patients were more likely to undergo delayed versus immediate closure in comparison to Hurley stage 2 patients ( *p* = 0.015). There were no statistically significant differences in the rate of postoperative complications when comparing the patients according to the Hurley stage ( Supplementary Table 4 , available in the online version).

## Discussion

The primary aim of this study was to examine the effects of both *method* and *timing* of post-HS excision reconstruction on postoperative outcome. Our first analysis focused on the timing of closure and compared immediate closure with delayed closure. We then performed a second analysis to assess the effects of any intervention (whether immediate or delayed) on postoperative outcome in comparison to secondary intention only.

**Fig. 1:**
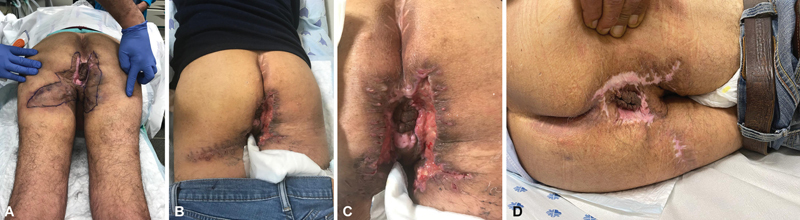
A 67-year-old man with Hurley stage 3 hidradenitis suppurativa (HS) involving the perineal region. ( **A** ) Extensive area of HS-affected skin in the perineal region. This was excised and closed primarily in an immediate fashion. ( **B, C** ) Partial wound breakdown treated with alginate dressing. ( **D** ) Following complete secondary intention.

Our study included 103 patients who underwent HS excision in 158 surgical sites. The overall postoperative complication rate was 25%, which is in line with previous publications. 2 9 10 Statistical analysis found that patients who underwent immediate closure had a statistically significant higher complication rate in comparison to those who underwent delayed closure (31 vs. 16%, *p* = 0.039). This was mainly due to a higher rate of wound breakdown (24 vs. 3%, *p* < 0.001) and may be explained in part by the nature of closure method as 90% of the cases in the immediate closure group were reconstructed using primary closure ( Fig. 1 ). Previous studies support the trend of higher complication rates when primary closure of post-HS excision wounds is used. 8 11 12 It has been postulated that this may be due to residual inflammatory HS-affected tissue, high contamination rates, and tension on wound edges that increase the risk of wound breakdown. 7 Of note, when comparing the two cohorts, there was no statistically significant difference in the rate of possible cofounders such as age, smoking, comorbidities, rate of multiple surgical areas per surgery, and the size of the defect. Statistically significant differences were noted, however, in mean BMI, Hurley stage, and rate of axillary surgeries, all of which were higher in the delayed closure cohort and may be considered risk factors for wound-related complications ( Table 2 ).

Our second analysis focused on the effects of reconstructive method on postoperative outcome while discarding the timing factor. This analysis showed that any type of intervention, in both the immediate and delayed settings, was involved with a statistically significant higher postoperative complication rate in comparison to secondary intention only (33 vs. 4%, *p* < 0.001; Supplementary Table 1 , available in the online version). With that said, mean time to wound closure in patients with secondary intention only was 85.4 days, and this prolonged time frame is in line with previous publications. 2 11 NPWT-assisted closure shortened this time period to a mean of 57 days, while patients who were treated with NPWT as a bridge for delayed STSG had the shortest time to wound closure with a mean of 22.5 days. This, however, was associated with increased risk of complications in comparison to secondary healing only ( Table 5 ). When analyzing these data, one must consider possible selection bias as treatment was tailored according to patient and wound characteristics (size, healing capacity, surrounding tissue properties, etc.).

Another aspect of our study was to explore potential risk factors for adverse postoperative outcome. Several factors emerged as risk factors for various adverse postoperative outcome and included age above 40 years, a large surface area of excision (above the mean for this cohort that was 8 cm ^2^ ), perioperative biologic treatment, and having at least one comorbidity. We therefore suggest that these factors be considered when creating a treatment plan.

It is commonly said that the only curative treatment for HS is wide surgical excision of affected skin. 13 14 This may involve localized excision of refractory nodules or more extensive procedures that involve excision of the entire hair-bearing skin of the affected region. 1 3 6 15 16 To date, there is no consensus regarding the best reconstructive approach for these patients. 9 17 Reconstructive goals are to provide closure of the surgical wound with the best possible aesthetic and functional outcome, while minimizing wound-related morbidity and interruption of daily activities. When considering reconstructive options following excision of HS, various options have been described in accordance with the “reconstructive ladder” and include secondary intention, immediate and delayed primary closure, skin grafting, local and regional flaps, and free flaps. 5 7 12 13 14 Small defects may be best dealt with secondary intention or primary closure, while larger defects may require skin grafting or flap closure. The timing and the proposed method of reconstruction will in turn affect decision-making regarding the extent of excision.

**Fig. 2:**
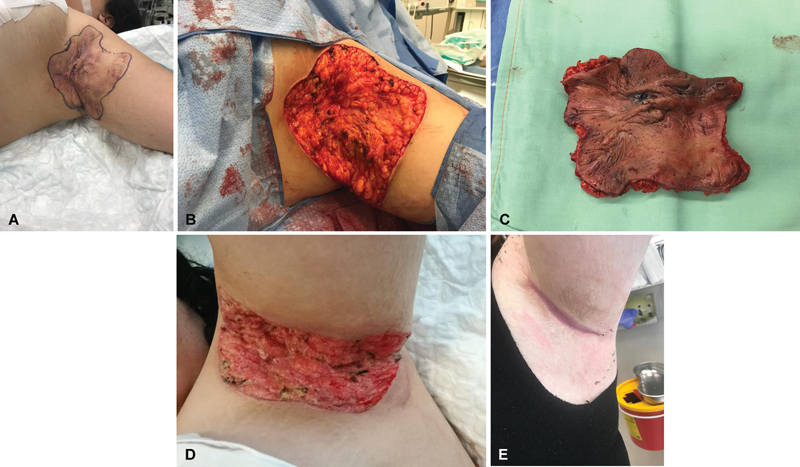
A 28-year-old woman with Hurley stage 3 hidradenitis suppurativa (HS) involving the bilateral axilla. ( **A** ) Left axilla showing severe scarring typical of HS. ( **B** ) Following complete excision of HS affected skin. ( **C** ) Excised tissue measuring 9 × 12 cm. ( **D** ) Following 2 weeks of negative pressure wound therapy (NPWT). ( **E** ) Complete wound closure following 12 weeks of NPWT.

**Fig. 3:**
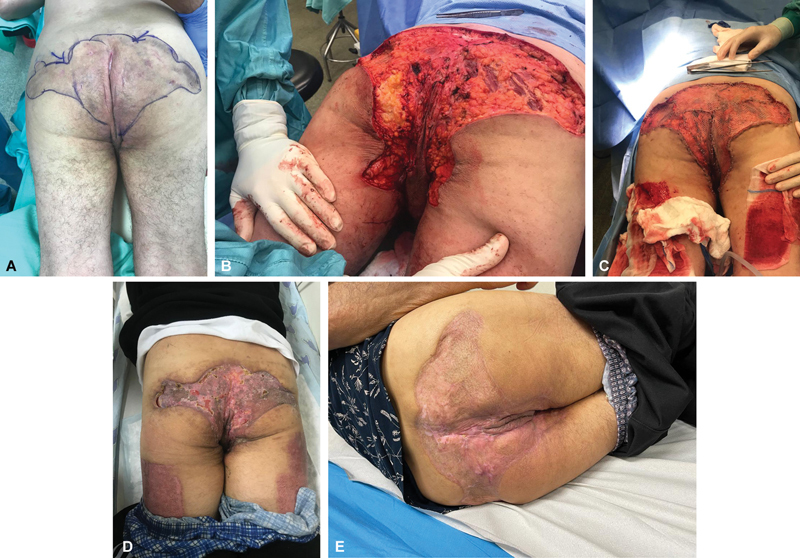
A 66-year-old man with extensive Hurley stage 3 hidradenitis suppurativa (HS) ( **A** ) Extensive HS disease. ( **B** ) Excision of HS affected skin and subcutaneous tissue. ( **C** ) Immediate meshed split-thickness skin graft (STSG). ( **D** ) 4-week postoperative result showing a mostly taken graft with foci of wound breakdown. ( **E** ) 12-week postoperative result showing stable graft and complete wound closure.

The findings of this study emphasize that the decision on *how* and *when* to reconstruct post-HS excision wounds is complex and multifactorial. Based on the findings of this study, and in accordance with our clinical experience, we recommend a delayed closure pathway for all patients, however certain parameters are taken into account and may change the treatment plan. Older patients with medical comorbidities, patients with a large excisional area, and patients with concurrent biologic treatment will probably benefit the most from a delayed closure approach. This may be done with the assistance of NPWT for larger wounds or by secondary intention only for smaller wounds, with or without foam or alginate dressings. This pathway may involve prolonged wound care, but is associated with a lower complication rate. Our experience shows that these wounds typically heal with a modest scar at the end of this course, even in large wounds ( Fig. 2 ). Cost-effectiveness of these modalities should be examined individually as costs of various topical agents and home-treatment plans may vary according to local health care systems and may affect decision-making. On the other hand, a young healthy patient with a small excisional procedure may benefit the most from immediate closure of his wound. This may allow for a rapid return to daily activities, but entails a higher chance of wound breakdown (OR of 2.26 in our cohort) that may cause a delay in recovery. All cases will require an individualized decision based on the pros and cons of each method ( Fig. 3 ).

Our study has several limitations. It is a retrospective study and thus suffers from its inherent limitations. The study did not include QOL and patient-reported outcome data. Our study examined the role of primary closure and skin grafting as the method of surgical intervention; flaps were not used and therefore conclusions on their role in the treatment of HS cannot be made according to the findings of this study. Finally, our study did not include cost assessment of these prolonged home-based treatment plans, and this warrants additional research to further assess cost-effectiveness of such modalities in this patient population.

## Conclusion

Our findings suggest that in HS surgery, delayed secondary intention leads to the most uneventful course in regard to perioperative adverse events; however, it involves a prolonged period of wound care. NPWT with delayed STSG shortens the time to wound closure; however, any intervention is associated with increased risk of complications, regardless of the timing of intervention.
