# Supplementary material for: Immediate versus Delayed Wound Closure in Hidradenitis Suppurativa Surgery: A Comparative Outcomes Study
Source: Indian J Plast Surg. 2025 Jan 31;58(3):191–8. doi: 10.1055/s-0045-1802326 (PMC12213030; doi:10.1055/s-0045-1802326)
Supplement: Supplementary file 1 — Supplementary Material [file 10-1055-s-0045-1802326-s2392412.pdf]

**Supplementary Table S1** Comparison of demographics, operative data, and postoperative outcome according to intervention type

| | Total | Immediate intervention | Delayed intervention | Any intervention | Secondary healing | *p*-Value immediate vs. delayed intervention | *p*-Value Any intervention vs. secondary healing |
|---|---|---|---|---|---|---|---|
| **Demographics** | | | | | | | |
| Patients | 69 | 44 | 8 | 51 | 28 | | |
| Surgeries | 103 | 61 | 13 | 73 | 36 | | |
| No. of regions | 158 | 91 | 21 | 112 | 46 | | |
| Gender | 89 (56%) | 46 (51%) | 14 (67%) | 60 (54%) | 29 (63%) | 0.185 | 0.278 |
| Hurley stage | 2.5 ± 0.6 | 2.4 ± 0.6 | 2.9 ± 0.3 | 2.5 ± 0.6 | 2.6 ± 0.5 | N/A | N/A |
| Follow-up (mo) | 11.6 ± 14.1 | 14.4 ± 15.5 | 10 ± 13.8 | 13.6 ± 15.2 | 7.0 ± 9.7 | N/A | N/A |
| MDT duration (mo) | 26 ± 19.2 | 28.5 ± 19 | 17.7 ± 18 | 26.4 ± 19.2 | 25.1 ± 19.4 | N/A | N/A |
| Prior biologic treatment | 77 (49%) | 39 (43%) | 19 (90%) | 58 (52%) | 19 (41%) | 0 | 0.234 |
| Current biologic treatment | 52 (33%) | 23 (25%) | 17 (81%) | 40 (36%) | 12 (26%) | 0 | 0.245 |
| Smoking | 92 (58%) | 52 (57%) | 12 (57%) | 64 (57%) | 28 (61%) | 1 | 0.669 |
| Comorbidities | 69 (44%) | 37 (41%) | 14 (67%) | 51 (46%) | 18 (39%) | 0 | 0.114 |
| Operative data | | | | | | | |
| Age at surgery (y) | 36.8 ± 13.4 | 37.6 ± 14.3 | 37.9 ± 11.7 | 37.7 ± 13.8 | 34.5 ± 12.3 | 0.935 | 0.18 |
| BMI at surgery | 26.8 ± 4.9 | 26 ± 4.8 | 26.7 ± 2.9 | 26.1 ± 4.5 | 28.4 ± 5.4 | 0.531 | 0.008 |
| Multiple operative sites | 103 (65%) | 65 (71%) | 12 (57%) | 77 (69%) | 26 (57%) | 0.206 | 0.145 |
| Total size of resection ($cm^2$) | 48 ± 64.7 | 48.9 ± 70.3 | 77.6 ± 85.1 | 54.3 ± 73.8 | 32.7 ± 29.2 | 0.108 | 0.056 |
| Surgery duration (min) | 52.9 ± 43.4 | 65.1 ± 50.3 | 43.6 ± 27.3 | 61.1 ± 47.5 | 33 ± 21 | 0.06 | 0 |
| **Postoperative outcome** | | | | | | | |
| Complications | 39 (25%) | 28 (31%) | 9 (43%) | 37 (33%) | 2 (4%) | 0.293 | 0 |
| Acute revision | 2 (1%) | 2 (2%) | 0 (0%) | 2 (2%) | 0 (0%) | 0.497 | 0.365 |
| Late revision (scar revision/ other) | 15 (9%) | 10 (11%) | 2 (10%) | 12 (11%) | 3 (7%) | 0.847 | 0.417 |
| Reoperation for recurrence | 32 (20%) | 21 (23%) | 2 (10%) | 23 (21%) | 9 (20%) | 0.169 | 0.891 |
| Hematoma | 5 (3%) | 2 (2%) | 2 (10%) | 4 (4%) | 1 (2%) | 0.105 | 0.651 |
| SSI | 11 (7%) | 8 (9%) | 2 (10%) | 10 (9%) | 1 (2%) | 0.916 | 0.131 |
| Seroma | 2 (1%) | 2 (2%) | 0 (0%) | 2 (2%) | 0 (0%) | 0.497 | 0.365 |
| Dehiscence | 24 (15%) | 22 (24%) | 2 (10%) | 24 (21%) | 0 (0%) | 0.143 | 0.001 |
| DVT | 0 (0%) | 0 (0%) | 0 (0%) | 0 (0%) | 0 (0%) | N/A | N/A |
| Other | 8 (5%) | 6 (7%) | 2 (10%) | 8 (7%) | 0 (0%) | 0.642 | 0.063 |

Abbreviations: BMI, body mass index; DVT, deep vein thrombosis; MDT, Multidisciplinary team; N/A, not applicable; SSI, surgical site infection.

 © 2025. The Author(s).

**Supplementary Table S2** Risk factors for reoperation and postoperative complications in the general study population: immediate reconstruction cohort

| | Reoperation/ recurrence | Late revision (scar revision/ other) | Complications | Hematoma | SSI | Dehiscence |
|---|---|---|---|---|---|---|
| Age >40 y at surgery | 0.8/0.8 | 0.69/0.74 | 1.73/0.25 | N/A/0.14 | 3.1/0.14 | 1.22/0.8 |
| BMI >25 at surgery | 0.81/0.8 | 0.08/0.01 | 0.74/0.65 | N/A/0.49 | 0.93/1.0 | 1.49/0.47 |
| Male gender | 1.41/0.62 | 1.54/0.74 | 1.47/0.5 | 0.0/0.24 | 0.98/1.0 | 1.24/0.81 |
| Multiple regions | 1.0/1.0 | 1.68/0.72 | 0.61/0.33 | N/A/1.0 | 1.22/1.0 | 0.47/0.18 |
| Total size of resection ($cm^2$) | 0.46/0.51 | 2.46/0.36 | 2.29/0.22 | N/A/0.03 | 6.55/0.02 | 1.74/0.51 |
| Prior biological treatment | 3.6/0.02 | 6.45/0.02 | 2.32/0.11 | N/A/0.18 | 11.16/0.02 | 1.87/0.22 |
| Current biological treatment | 0.63/0.57 | 2.18/0.27 | 0.98/1.0 | N/A/0.06 | 6.02/0.02 | 0.83/1.0 |
| Smoking | 1.68/0.45 | 3.36/0.18 | 1.92/0.25 | 0.0/0.18 | 0.73/0.72 | 1.85/0.32 |
| Comorbidities[a] | 2.44/0.11 | 3.7/0.11 | 2.53/0.11 | N/A/1.0 | 6.36/0.11 | 1.3/0.78 |

Abbreviations: BMI, body mass index; OR, odds ratio; SSI, surgical site infection.
Note: OR and *p*-value were calculated using the SciPy.stats module for Python.
*Statistically significant.
[a]One or more of the following: inflammatory bowel disease, diabetes mellitus, asthma, dyslipidemia, hypertension, ischemic heart disease, rheumatoid arthritis, and familial Mediterranean fever.

**Supplementary Table S3** Risk factors for reoperation and postoperative complications in the general study population: delayed reconstruction cohort

| | Reoperation/ recurrence | Late revision (scar revision/ other) | Complications | Hematoma | SSI | Wound breakdown |
|---|---|---|---|---|---|---|
| Age >40 y at surgery | 0.0/0.01 | 1.51/0.65 | 5.25/0.03 | 0.0/0.55 | 1.1/1.0 | 2.25/0.53 |
| BMI >25 at surgery | 0.62/0.51 | 0.82/1.0 | 0.97/1.0 | 0.26/0.29 | 0.26/0.29 | N/A/0.53 |
| Male gender | 0.62/0.51 | N/A/0.15 | 2.91/0.3 | 1.12/1.0 | N/A/0.55 | 0.55/1.0 |
| Multiple regions | 4.19/0.1 | 3.29/0.38 | 4.19/0.1 | N/A/0.25 | N/A/0.25 | 0.76/1.0 |
| Total size of resection ($cm^2$) | 0.0/0.58 | 3.62/0.33 | 31.43/0.0 | 0.0/1.0 | 0.0/1.0 | 15.25/0.14 |
| Prior biological treatment | 2.31/0.33 | N/A/0.06 | 2.31/0.33 | 1.56/1.0 | 1.56/1.0 | 0.76/1.0 |
| Current biological treatment | 1.72/0.51 | 0.86/1.0 | 4.44/0.05 | 2.74/0.57 | 2.74/0.57 | 1.32/1.0 |
| Smoking | 0.5/0.33 | 0.15/0.15 | 0.78/0.75 | 1.37/1.0 | N/A/0.27 | N/A/0.51 |
| Comorbidities[a] | 2.77/0.17 | 4.0/0.28 | 0.91/1.0 | 0.0/0.54 | 0.92/1.0 | 0.0/1.0 |

Abbreviations: BMI, body mass index; OR, odds ratio; SSI, surgical site infection.
Note: OR and *p*-value were calculated using SciPy.stats module for Python.
*Statistically significant.
[a]One or more of the following: inflammatory bowel disease, diabetes mellitus, asthma, dyslipidemia, hypertension, ischemic heart disease, rheumatoid arthritis, and familial Mediterranean fever.

 © 2025. The Author(s).

**Supplementary Table S4** Comparison of demographics, operative data, and postoperative outcome according to Hurley stage

| | Total | Hurley stage 1 | Hurley stage 2 | Hurley stage 3 | *p*-Value 1:2 | *p*-Value 1:3 | *p*-Value 2:3 |
|---|---|---|---|---|---|---|---|
| Patients | 69 | 6 | 32 | 32 | | | |
| Surgeries | 103 | 6 | 41 | 56 | | | |
| No. of regions | 158 | 9 | 59 | 90 | | | |
| Gender (male) | 89 (56%) | 4(44%) | 26 (44%) | 59 (66%) | 0.983 | 0.983 | 0.213 |
| Hurly stage | 2.5 ± 0.6 | 1 ± 0 | 2 ± 0 | 3 ± 0 | 0 | 0 | 0 |
| Follow-up (mo) | 11.6 ± 14 | 8.4 ± 7.1 | 12.3 ± 17.6 | 11.5 ± 11.8 | 0.524 | 0.524 | 0.444 |
| MDT duration | 26 ± 19.1 | 21.0 ± 16.7 | 22.4 ± 19.4 | 29.0 ± 18.8 | 0.841 | 0.841 | 0.224 |
| Prior ABX | 155 (98%) | 9 (100%) | 56 (95%) | 90 (100%) | 0.496 | 0.496 | N/A |
| Prior IMMUNE | 77 (49%) | 1 (11%) | 9 (15%) | 67 (74%) | 0.748 | 0.748 | 0 |
| Current IMMUNE | 52 (33%) | 1 (11%) | 5 (8%) | 46 (51%) | 0.799 | 0.799 | 0.022 |
| Smoking | 92 (58%) | 1 (11%) | 38 (64%) | 53 (59%) | 0.002 | 0.002 | 0.006 |
| Comorbidities | 69 (44%) | 5 (56%) | 20 (34%) | 44 (49%) | 0.215 | 0.215 | 0.39 |
| Age at surgery | 36.8 ± 13.4 | 29.9 ± 13.8 | 33.4 ± 12.5 | 39.7 ± 13.4 | 0.442 | 0.442 | 0.04 |
| BMI at surgery | 26.8 ± 4.9 | 24.2 ± 5.67 | 26.1 ± 4.9 | 27.5 ± 4.7 | 0.284 | 0.284 | 0.048 |
| Multiple regions | 103 (65%) | 6 (67%) | 36 (61%) | 61 (68%) | 0.75 | 0.75 | 0.947 |
| Total size of resection ($cm^2$) | 48 ± 64.7 | 19.7 ± 16.6 | 32.7 ± 43.8 | 60.9 ± 75.6 | 0.386 | 0.386 | 0.108 |
| Total size of resection ($cm^3$) | 125.2 ± 221.6 | 22.6 ± 10.4 | 90.5 ± 231.1 | 152.9 ± 221 | N/A | N/A | N/A |
| Operation duration (min) | 52.9 ± 43.4 | 33.3 ± 22.2 | 38.5 ± 20.8 | 64.3 ± 51.9 | 0.494 | 0.494 | 0.08 |
| Operation admission duration | 10.2 ± 16.56 | 2.7 ± 1.7 | 2.8 ± 2.6 | 15.9 ± 20.1 | 0.914 | 0.914 | 0.052 |
| Immediate | 91 (58%) | 8 (89%) | 41 (69%) | 42 (47%) | 0.233 | 0.233 | 0.015 |
| Delayed | 67 (42%) | 1 (11%) | 18 (31%) | 48 (53%) | 0.233 | 0.233 | 0.015 |
| Primary closure | 85 (54%) | 8 (89%) | 41 (69%) | 36 (40%) | 0.233 | 0.233 | 0.005 |
| STSG | 27 (17%) | 0 (0%) | 2 (3%) | 25 (28%) | 0.582 | 0.582 | 0.069 |
| Secondary intention | 46 (29%) | 1 (11%) | 16 (27%) | 29 (32%) | 0.309 | 0.309 | 0.193 |
| Complications | 39 (25%) | 3 (33%) | 13 (22%) | 23 (26%) | 0.464 | 0.464 | 0.617 |
| Acute revision | 2 (1%) | 0 (0%) | 0 (0%) | 2 (2%) | N/A | N/A | 0.655 |
| Late revision (scar revision/other) | 15 (9%) | 0 (0%) | 2 (3%) | 13 (14%) | 0.582 | 0.582 | 0.225 |
| Reoperation/ recurrence | 32 (20%) | 0 (0%) | 9 (15%) | 23 (26%) | 0.214 | 0.214 | 0.085 |
| Hematoma | 5 (3%) | 0 (0%) | 0 (0%) | 5 (6%) | N/A | N/A | 0.473 |
| SSI | 11 (7%) | 0 (0%) | 3 (5%) | 8 (9%) | 0.496 | 0.496 | 0.356 |
| Seroma | 2 (1%) | 1 (11%) | 1 (2%) | 0 (0%) | 0.123 | 0.123 | 0.001 |
| Dehiscence | 24 (15%) | 1 (11%) | 12 (20%) | 11 (12%) | 0.519 | 0.519 | 0.923 |
| DVT | 0 (0%) | 0 (0%) | 0 (0%) | 0 (0%) | N/A | N/A | N/A |
| Other | 8 (5%) | 2 (22%) | 0 (0%) | 6 (7%) | 0 | 0 | 0.105 |
| Groin | 61 (39%) | 3 (33%) | 31 (53%) | 27 (30%) | 0.29 | 0.29 | 0.838 |
| Axilla | 52 (33%) | 3 (33%) | 16 (27%) | 33 (37%) | 0.704 | 0.704 | 0.845 |
| Buttocks | 12 (8%) | 0 (0%) | 4 (7%) | 8 (9%) | 0.428 | 0.428 | 0.356 |
| Perineal | 15 (9%) | 0 (0%) | 3 (5%) | 12 (13%) | 0.496 | 0.496 | 0.247 |
| Breast | 8 (5%) | 2 (22%) | 2 (3%) | 4 (4%) | 0.025 | 0.025 | 0.033 |
| Other | 10 (6%) | 1 (11%) | 3 (5%) | 6 (7%) | 0.482 | 0.482 | 0.624 |

Abbreviations: ABX, antibiotic; BMI, body mass index; DVT, deep vein thrombosis; STSG, split-thickness skin graft; N/A, not applicable; SSI, surgical site infection.

 © 2025. The Author(s).
